# Association of Obesity With Mortality Over 24 Years of Weight History: Findings From the Framingham Heart Study

**DOI:** 10.1001/jamanetworkopen.2018.4587

**Published:** 2018-11-16

**Authors:** Hanfei Xu, L. Adrienne Cupples, Andrew Stokes, Ching-Ti Liu

**Affiliations:** 1 Department of Biostatistics, Boston University School of Public Health, Boston, Massachusetts; 2 National Heart, Lung, and Blood Institute and Boston University Framingham Heart Study, Framingham, Massachusetts; 3 Department of Global Health, Boston University School of Public Health, Boston, Massachusetts

## Abstract

**Importance:**

Many studies of the association between obesity and mortality rely on weight status at a single point in time, making it difficult to adequately address bias associated with reverse causality.

**Objective:**

To investigate the association between maximum body mass index (BMI) and all-cause mortality without the consequences of reverse causality.

**Design, Setting, and Participants:**

Prospective cohort studies for the original and offspring cohorts of the Framingham Heart Study. The follow-up period started from baseline examination 13 for the original cohort and from baseline examination 6 for the offspring cohort and ended December 31, 2014. The analyses were conducted in 2017. Participants were 6197 individuals with 3478 deaths during a mean of 17 years of follow-up.

**Main Outcomes and Measures:**

Maximum BMI over 24 years of weight history before the beginning of follow-up for all-cause mortality and cause-specific mortality. All-cause mortality and cause-specific mortality (deaths due to cardiovascular disease, cancer, or other causes).

**Results:**

Among 6197 participants (mean [SD] age at baseline, 62.79 [8.98] years; 55.5% female), 3478 (56.1%) died during the follow-up. A monotonic association was observed between maximum BMI and mortality, with increasing risks observed across obese I (BMI of 30 to <35; hazard ratio [HR], 1.27; 95% CI, 1.14-1.41) and obese II (BMI of 35 to <40; HR, 1.93; 95% CI, 1.68-2.20) categories. A significant association was not observed for the overweight category (BMI of 25 to <30; HR, 1.08; 95% CI, 0.99-1.18). Among never smokers, the risks increased, with a significant association emerging for individuals with maximum BMI in the overweight range (HR, 1.31; 95% CI, 1.13-1.51). The mortality rates of normal-weight individuals who were formerly overweight or obese were 47.48 and 66.67 per 1000 person-years, respectively, while individuals who never exceeded normal weight had a mortality rate of 27.93 per 1000 person-years.

**Conclusions and Relevance:**

A monotonic association was found between maximum BMI over 24 years of weight history and subsequent all-cause mortality. Maximum BMI in the normal-weight range was associated with the lowest risk of mortality in this cohort, highlighting the importance of obesity prevention.

## Key Points

**Question:**

Is there an association between obesity and mortality incorporating weight history?

**Findings:**

In this cohort study that included 6197 participants from the original and offspring cohorts of the Framingham Heart Study, a monotonic association was observed between maximum body mass index measured over 24 years of weight history and subsequent mortality, with increasing risks observed across the obese I and obese II categories compared with the normal-weight group.

**Meaning:**

Eliciting weight history in clinical practice may be valuable for identifying patients at increased risk of mortality.

## Teaser

This cohort study investigates the association between maximum body mass index (BMI) and all-cause mortality for the original and offspring cohorts of the Framingham Heart Study without the consequences of reverse causality.

## Introduction

Numerous investigations of the association between obesity and all-cause mortality have been conducted.^1,2,3,4,5,6,7,8^ A major potential threat to the validity of such studies is the issue of confounding by illness, in which a preexisting condition alters both weight status and the risk of mortality.^9,10^ This source of bias, also referred to as reverse causality, has been cited^8^ as a potential explanation for the findings of a 2013 meta-analysis^11^ of 97 studies among more than 2.88 million individuals. In that meta-analysis compared with normal weight (body mass index [BMI] of 18.5 to <25, calculated as weight in kilograms divided by height in meters squared), overweight (BMI of 25 to <30) was associated with lower all-cause mortality, and obese I (BMI of 30 to <35) was not associated with higher mortality.

In a subsequent meta-analysis^8^ based on more than 10 million participants from 239 prospective studies, the Global BMI Mortality Collaboration generated estimates of obesity-mortality associations in a series of restricted analyses in which various criteria were used to mitigate the risk of reverse causal bias. These strategies included delaying the beginning of analysis until several years after study entry and eliminating individuals with a preexisting disease at entry. A major shortcoming of those strategies is that they trigger severe exclusions of data, resulting in losses of precision and generalizability.^12^ Also, preclinical and undiagnosed diseases are ignored in these approaches; as such, they do not represent a comprehensive solution to addressing bias associated with illness-induced weight loss. Furthermore, diseases may be caused by obesity itself, so excluding people with a disease at baseline could lead to overadjustment and result in attenuated associations.^13^

In the setting of a prospective study, using maximum BMI before the beginning of survival follow-up instead of BMI at entry to define an individual’s BMI status is a recently proposed method that may mitigate reverse causality without sample exclusions. Several prior studies^14,15,16^ have used maximum BMI to distinguish people belonging to the normal BMI category over time from people who transitioned into the normal BMI category as a result of weight loss caused by illness. Investigators using this approach consistently found that maximum BMI in the overweight, obese I, and obese II (BMI of 35 to <40) categories was associated with increases in risk for all-cause mortality. Among these studies, Stokes^14^ and Stokes and Preston^16^ constructed maximum BMI based on respondents’ recall of their maximum lifetime weight in the National Health and Nutrition Examination Survey (NHANES), possibly leading to recall bias. Yu et al^15^ performed a study on 3 large cohorts (Nurses’ Health Study I and II and Health Professionals Follow-Up study) with biennially updated health-related information. Because these longitudinal BMIs are self-reported, systematic underestimation of BMI and misclassification may exist, possibly overstating hazard ratios (HRs) of higher BMI categories (obese I and obese II).^17^ This process may be misleading for other BMI categories, especially when the reference category is altered by misreporting.^18^

To address these limitations, we investigated the association between maximum BMI and all-cause mortality using data from the original and offspring cohorts of the Framingham Heart Study (FHS). The objective of our study was to produce risk estimates based on maximum BMI defined from longitudinally measured weight and height before the beginning of follow-up rather than self-reported values. These measured values are expected to improve the accuracy of BMI data and thus lead to better estimates of the association between obesity and mortality.

## Methods

### Study Population

We performed prospective cohort studies for the original and offspring cohorts of the FHS. The design and selection criteria of the original FHS^19^ and the Framingham Offspring Study^20^ have been described previously. The original cohort consisted of 5209 participants of a two-thirds systematic sample of the population in Framingham, Massachusetts, aged 28 to 62 years in 1948. The offspring cohort of 5124 participants, aged 20 to 59 years at entry, was initiated in 1971 to establish a prospective epidemiologic study of the offspring of the original cohort and the offspring spouses. Examinations were performed every 2 years for the original cohort and every 4 to 8 years for the offspring cohort to follow up with their health-related information. Participants provided written informed consent, and the Boston University Medical Center Institutional Review Boards approved the study. We followed Strengthening the Reporting of Observational Studies in Epidemiology (STROBE) reporting guideline for observational studies to guide the reporting of this study.

### Measurements

#### Body Mass Index

Body weight in pounds and height in inches were measured at every examination, and BMI was calculated for each examination. Body mass index was categorized into the following predefined categories: underweight (<18.5), normal weight (18.5 to <25), overweight (25 to <30), obese I (30 to <35), and obese II (35 to <40).^21^ Normal weight was treated as the reference category in our analyses.

#### Baseline Examination

**Figure 1.**
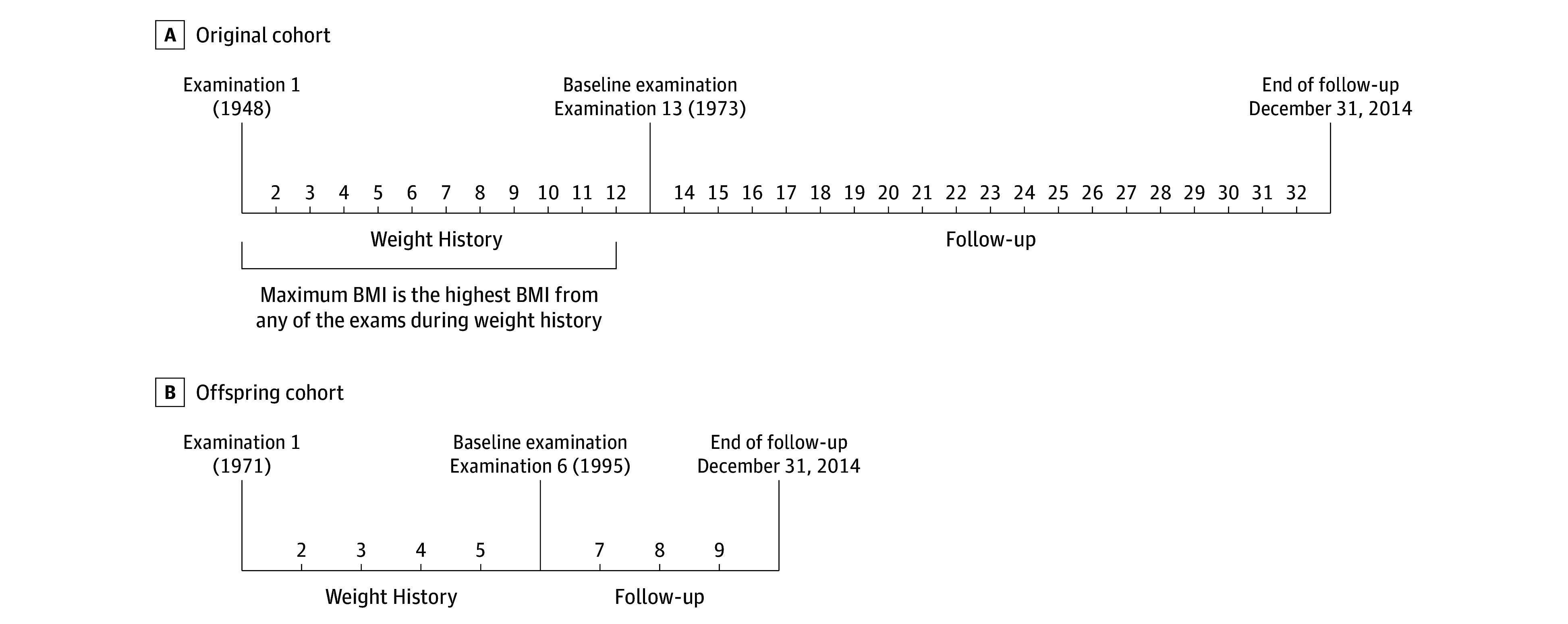
Diagram of the Study Design Shown are the original cohort (A) and offspring cohort (B) of the Framingham Heart Study. BMI indicates body mass index.

We defined the baseline examination (the beginning of survival follow-up) as examination 13 for the original cohort and examination 6 for the offspring cohort (Figure 1). We chose these examinations to estimate maximum BMI before the baseline examination and to have sufficient survival follow-up to evaluate mortality risk after the baseline examination. The follow-up period for each participant started from the date of baseline examination 13 (conducted between 1973 and 1975) for the original cohort and from the date of baseline examination 6 (conducted between 1995 and 1998) for the offspring cohort and ended at death, age 95 years, or December 31, 2014, whichever occurred first. The analyses were conducted in 2017.

#### Maximum BMI

The weight history period of each participant was defined as the interval between examination 1 and the baseline examination. Maximum BMI was captured by taking the highest BMI during the weight history period. We considered a weight history length of 24 years for both the original and offspring cohorts so that we had sufficient weight history observations and follow-up time.

#### Annual BMI Loss

We defined annual weight loss by subtracting BMI at the baseline examination from maximum BMI and then dividing by duration (in years) between the examination of reaching maximum BMI and the baseline examination. We used the 75th percentile of the distribution to dichotomize annual BMI loss into slow vs rapid, excluding participants whose maximum BMI was equal to their BMI at the baseline examination.

### Sample Restriction

Participants who died or were lost to follow-up before the baseline examination were excluded, and the sample was restricted to adults 20 years or older at time of entry (1948 for the original cohort and 1971 for the offspring cohort). We only included participants who had 3 or more weight records during the weight history period and excluded people who did not have a weight measured at the baseline examination. We excluded participants belonging to the underweight category at baseline because of small sample sizes. We did not exclude smokers or participants with baseline illness.

All-cause mortality after the baseline examination was the primary outcome of our study. Participants were followed up to 42 years. Covariates included the following: cohort (original or offspring), sex, smoking status (never, former, or current), menopause status (yes or no) if female, alcohol consumption (in ounces per week), duration (in years) between the examination of reaching maximum BMI and the baseline examination, prior cardiovascular disease (CVD) (yes or no), and prior cancer (yes or no). For individuals with missing covariate information at baseline, we carried forward values from the previous examination if available and excluded them from the analysis otherwise. A detailed sample exclusion flowchart is shown in eFigure 1 in the Supplement.

### Statistical Analysis

We used FHS data collected from 1948 to 2014. We conducted Cox proportional hazards regression analyses to estimate HRs over the follow-up time from the baseline examination and their corresponding 95% CIs associated with each BMI category compared with the reference normal-weight category. We adjusted for cohort, age at baseline, sex, smoking status, menopause status, alcohol consumption, and duration between the examination of reaching maximum BMI and the baseline examination. Maximum BMI was the primary measurement of interest and was not updated during follow-up. Models were estimated for all individuals and separately for never smokers. Stratified analyses by sex were also performed. Additional analyses were performed in which maximum BMI was specified as a continuous variable for all participants and separately for participants with maximum BMI of 25 or higher. We also assessed whether there was a significant interaction between maximum BMI categories and cohort to evaluate if the magnitude of the association between maximum BMI and mortality differed between the original and offspring cohorts. We performed age-stratified analyses using age 70 years as a cutoff point. Cohort-stratified analyses were performed as well. Cause-specific mortality (deaths due to CVD, cancer, or other causes, separately) was treated as the outcome in secondary analyses. We also performed analyses on the association between dichotomized annual BMI loss (slow vs rapid) and all-cause mortality, excluding people whose maximum BMI was equal to BMI at baseline. The proportional hazards assumption for maximum BMI was satisfied for both samples of all participants and never smokers. Sensitivity analyses were conducted by further adjusting for BMI at the baseline examination. Analyses were conducted with statistical software (SAS, version 9.4; SAS Institute Inc) using a 2-tailed α level of .05.

## Results

### Study Participants

Table 1 lists baseline characteristics at the beginning of survival follow-up (examination 13 for the original cohort and examination 6 for the offspring cohort) for the combined sample of 6197 participants. A total of 3478 deaths (56.1%) occurred during a mean of 17 years of follow-up over 105 210 person-years. The mean (SD) age at baseline of the sample was 62.79 (8.98) years, and 55.5% were female. The offspring cohort (mean [SD] age, 59.78 [8.97] years) was younger than the original cohort (mean [SD] age, 66.16 [7.71] years). There was little difference in the mean ages between men (mean [SD] age, 62.47 [8.71] years) vs women (mean [SD] age, 63.04 [9.18] years). Current smokers (mean [SD] age, 60.62 [7.65] years) were younger than never smokers (mean [SD] age, 63.88 [9.29] years) and former smokers (mean [SD] age, 62.41 [8.97] years) at baseline. The original cohort had a higher proportion of current smokers than the offspring cohort (25.2% [737 of 2922] vs 14.7% [483 of 3275]). Men and current smokers had higher alcohol consumption than other groups.

**Table 1.** Baseline Characteristics for the Original and Offspring Cohorts of the Framingham Heart Study^a^

Variable	All	Cohort		Sex	
		Original	Offspring	Male	Female
All	6197	2922	3275	2758	3439
Age at baseline, mean (SD), y	62.79 (8.98)	66.16 (7.71)	59.78 (8.97)	62.47 (8.71)	63.04 (9.18)
Maximum BMI before the baseline examination, No. (%)					
Normal	1404 (22.7)	643 (22.0)	761 (23.2)	349 (12.7)	1055 (30.7)
Overweight	2908 (46.9)	1488 (50.9)	1420 (43.4)	1506 (54.6)	1402 (40.8)
Obese I	1337 (21.6)	588 (20.1)	749 (22.9)	705 (25.6)	632 (18.4)
Obese II	548 (8.8)	203 (6.9)	345 (10.5)	198 (7.2)	350 (10.2)
Baseline BMI, No. (%)					
Normal	2079 (33.5)	1095 (37.5)	984 (30.0)	679 (24.6)	1400 (40.7)
Overweight	2689 (43.4)	1324 (45.3)	1365 (41.7)	1409 (51.1)	1280 (37.2)
Obese I	1043 (16.8)	395 (13.5)	648 (19.8)	528 (19.1)	515 (15.0)
Obese II	386 (6.2)	108 (3.7)	278 (8.5)	142 (5.1)	244 (7.1)
Sex, No. (%)					
Male	2758 (44.5)	1229 (42.1)	1529 (46.7)	2758 (100)	NA
Female	3439 (55.5)	1693 (57.9)	1746 (53.3)	NA	3439 (100)
Smoking status, No. (%)					
Never	3075 (49.6)	1282 (43.9)	1793 (54.7)	1220 (44.2)	1855 (53.9)
Former	1902 (30.7)	903 (30.9)	999 (30.5)	1011 (36.7)	891 (25.9)
Current	1220 (19.7)	737 (25.2)	483 (14.7)	527 (19.1)	693 (20.2)
Menopause status, No. (%)					
Missing (male)	2758 (44.5)	1229 (42.1)	1529 (46.7)	2758 (100)	NA
Yes	2696 (43.5)	1687 (57.7)	1009 (30.8)	NA	2696 (78.4)
No	743 (12.0)	6 (0.2)	737 (22.5)	NA	743 (21.6)
Alcohol consumption, mean (SD), oz/wk	2.90 (4.45)	3.48 (5.15)	2.38 (3.63)	4.31 (5.62)	1.78 (2.74)
Duration between the examination of reaching maximum BMI and the baseline examination, mean (SD), y	6.37 (7.48)	7.94 (7.84)	4.98 (6.85)	6.91 (7.57)	5.95 (7.38)
Follow-up time, mean (SD), y	16.98 (7.75)	18.32 (10.21)	15.78 (4.22)	15.68 (7.54)	18.02 (10.70)

Abbreviations: BMI, body mass index; NA, not applicable.

^a^ Body mass index (calculated as weight in kilograms divided by height in meters squared) is categorized as normal (18.5 to <25), overweight (25 to <30), obese I (30 to <35), or obese II (35 to <40). The baseline examination for the original cohort was examination 13 (1973-1975), and the baseline examination for the offspring cohort was examination 6 (1995-1998) and was the beginning of survival follow-up.

Using maximum BMI, 77.3% (4793 of 6197) of participants were overweight or obese, while 66.5% (4118 of 6197) were overweight or obese using baseline BMI. A higher proportion of women than men had maximum BMI in normal (30.7% [1055 of 3439] vs 12.7% [349 of 2758]) and obese II (10.2% [350 of 3439] vs 7.2% [198 of 2758]) categories. The number of years between the examination of reaching maximum BMI and the baseline examination was larger in the original cohort (mean [SD], 7.94 [7.84] years) than in the offspring cohort (mean [SD], 4.98 [6.85] years). Detailed characteristics of prior conditions across BMI categories are listed in eTable 1 in the Supplement. Generally, people with weight loss were more likely to have had a prior diagnosis of CVD or cancer than people who remained in the same weight category.

### All-Cause Mortality

**Figure 2.**
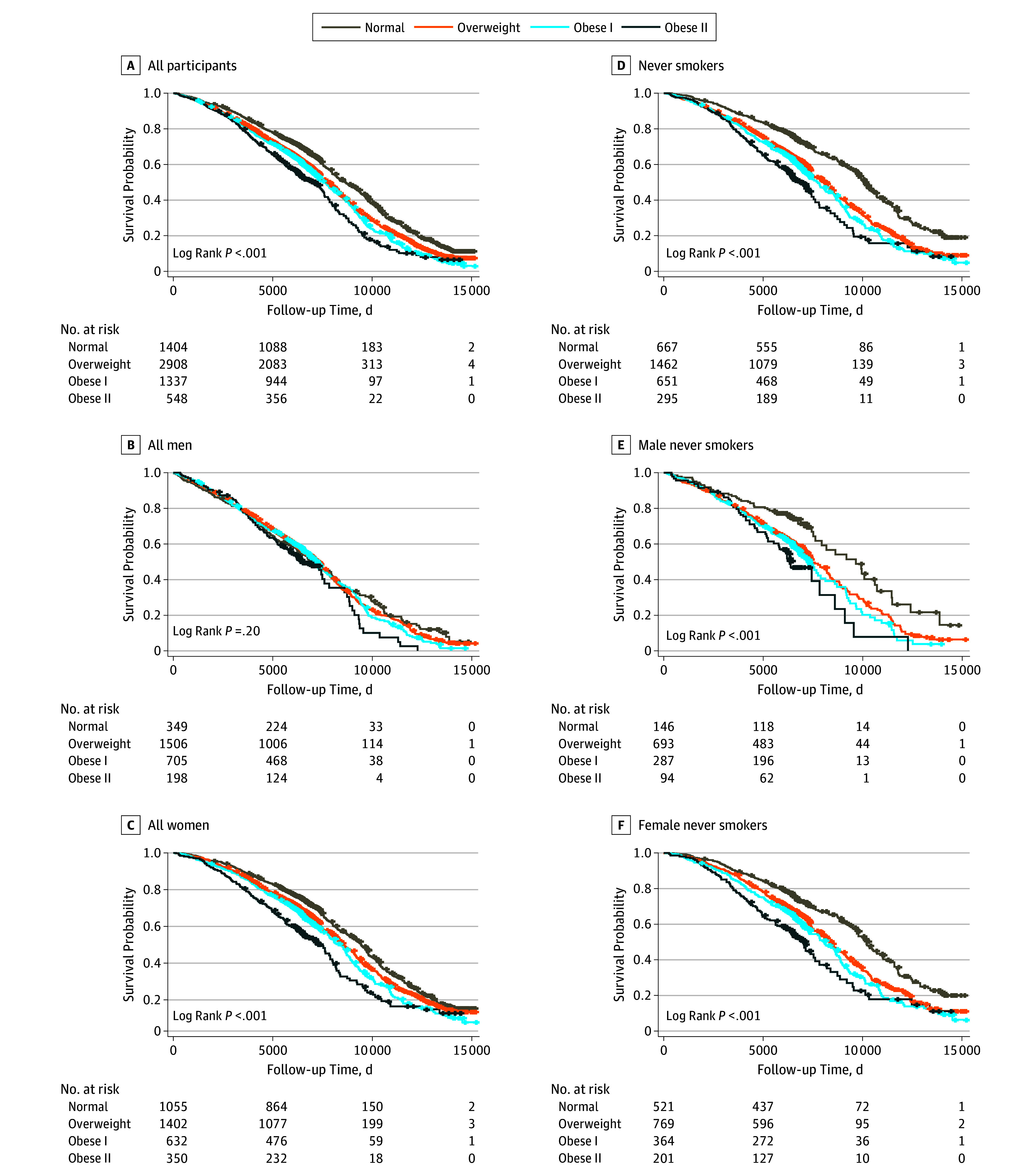
Kaplan-Meier Curves for Categories of Maximum BMI For numbers at risk, maximum body mass index (BMI; calculated as weight in kilograms divided by height in meters squared) is categorized as normal (18.5 to <25), overweight (25 to <30), obese I (30 to <35), or obese II (35 to <40).

For all participants, there was an upward trend of HRs from normal weight to obese II, indicating an increasing mortality risk with higher maximum BMI (Table 2). We observed significant associations between maximum BMI and all-cause mortality in the obese I (HR, 1.27; 95% CI, 1.14-1.41) and obese II (HR, 1.93; 95% CI, 1.68-2.20) categories; the overweight group also had an HR above 1 (1.08; 95% CI, 0.99-1.18) but it was not statistically significant. When stratified by sex, men had larger risk in overweight, obese I, and obese II categories than women. Kaplan-Meier curves by maximum BMI category (Figure 2A-C) revealed differences in survival probabilities across BMI categories in both the full sample and the female sample.

**Table 2.** Hazard Ratios for All-Cause Mortality in the Original and Offspring Cohorts of the Framingham Heart Study for Categories of Maximum BMI With 24 Years of Weight History, Stratified by Sex and Smoking Status^a^

Variable	Categorized Maximum BMI				Continuous Maximum BMI	
	Normal	Overweight	Obese I	Obese II	Per 5-U Increase	Per 5-U Increase (≥25)
**All Individuals**						
No. of events	710	1692	763	313	3478	2767
Person-years, No. (in thousands)	25.42	49.63	21.88	8.28	105.21	79.78
Multivariable HR (95% CI)	1 [Reference]	1.08 (0.99-1.18)	1.27 (1.14-1.41)	1.93 (1.68-2.20)	1.22 (1.17-1.27)	1.27 (1.21-1.33)
Male						
No. of events	214	923	418	116	1671	1457
Person-years, No. (in thousands)	5.55	23.93	10.91	2.86	43.25	37.70
Multivariable HR (95% CI)	1 [Reference]	1.16 (0.99-1.34)	1.38 (1.16-1.63)	2.39 (1.89-3.01)	1.30 (1.21-1.39)	1.37 (1.27-1.48)
Female						
No. of events	496	769	345	197	1807	1310
Person-years, No. (in thousands)	19.87	25.70	10.97	5.43	61.96	42.08
Multivariable HR (95% CI)	1 [Reference]	1.05 (0.94-1.18)	1.24 (1.08-1.43)	1.77 (1.50-2.10)	1.19 (1.14-1.25)	1.24 (1.17-1.31)
**Never Smokers**						
No. of events	258	757	355	164	1534	1275
Person-years, No. (in thousands)	12.42	24.87	10.79	4.47	52.55	40.11
Multivariable HR (95% CI)	1 [Reference]	1.31 (1.13-1.51)	1.57 (1.34-1.85)	2.38 (1.95-2.90)	1.29 (1.23-1.36)	1.30 (1.22-1.39)
Male						
No. of events	57	359	153	53	622	565
Person-years, No. (in thousands)	2.59	10.98	4.45	1.37	19.39	16.79
Multivariable HR (95% CI)	1 [Reference]	1.77 (1.33-2.34)	2.10 (1.54-2.86)	3.85 (2.62-5.65)	1.49 (1.34-1.66)	1.46 (1.29-1.65)
Female						
No. of events	201	398	202	111	912	710
Person-years, No. (in thousands)	9.84	13.89	6.34	3.10	33.16	23.31
Multivariable HR (95% CI)	1 [Reference]	1.17 (0.98-1.38)	1.43 (1.17-1.74)	2.02 (1.60-2.55)	1.24 (1.16-1.32)	1.25 (1.15-1.35)

Abbreviations: BMI, body mass index; HR, hazard ratio.

^a^ Body mass index (calculated as weight in kilograms divided by height in meters squared) is categorized as normal (18.5 to <25), overweight (25 to <30), obese I (30 to <35), or obese II (35 to <40). The multivariable model includes maximum BMI (categorized or continuous) and other covariates, including cohort, age at baseline, sex (if appropriate), smoking status (if appropriate), menopause status (if appropriate), alcohol consumption, and duration between the examination of reaching maximum BMI and the baseline examination.

The results for continuous maximum BMI (Table 2) showed similar trends. Specifically, for all participants, the HR (1.22; 95% CI, 1.17-1.27) suggested a 21.9% increased risk with each 5-unit increase in maximum BMI. Also, men had larger HRs than women. Restricting analysis to people with maximum BMI of 25 or higher, the HR (1.27; 95% CI, 1.21-1.33) was larger than in the full sample. Hazard ratios for men and women also increased in this subsample.

For people who belonged to overweight and obese I categories in the full sample and in men, the offspring cohort had significantly lower risk of mortality compared with the original cohort (HR, 0.81; 95% CI, 0.66-0.99 for overweight; and HR, 0.72; 95% CI, 0.57-0.91 for obese I) (eTable 2 in the Supplement). Cohort-specific results are summarized in eTable 3 in the Supplement. In age-stratified analyses (eTable 4 in the Supplement), HRs became weaker above age 70 years compared with below age 70 years using baseline BMI for the obese I (HR, 1.23; 95% CI, 1.08-1.40 for age <70 years; HR, 1.19; 95% CI, 0.99-1.44 for age ≥70 years) and obese II (HR, 1.95; 95% CI, 1.62-2.34 for age <70 years; HR, 1.51; 95% CI, 1.10-2.07 for age ≥70 years) categories. However, in the model using maximum BMI, we observed the opposite pattern, with the HRs strengthening above age 70 years for obese I (HR, 1.20; 95% CI, 1.06-1.37 for age <70 years; HR, 1.48; 95% CI, 1.22-1.78 for age ≥70 years) and obese II (HR,1.92; 95% CI, 1.63-2.25 for age <70 years; HR, 2.06; 95% CI, 1.60-2.65 for age ≥70 years) categories.

Hazard ratios were larger when we further adjusted for BMI at the baseline examination (HR, 1.20; 95% CI, 1.08-1.34 for overweight; HR, 1.59; 95% CI, 1.35-1.87 for obese I; and HR, 2.84, 95% CI, 2.20-3.67 for obese II) (details are listed in eTable 5 in the Supplement). In analyses of the association between dichotomized annual BMI loss and all-cause mortality, people with rapid BMI loss (HR, 1.36; 95% CI, 1.23-1.50) had a higher HR than people with slow BMI loss.

Among never smokers (n = 3075), a total of 1534 deaths were observed during 52 550 person-years of follow-up. Hazard ratios for overweight (HR, 1.31; 95% CI, 1.13-1.51), obese I (HR, 1.57; 95% CI, 1.34-1.85), and obese II (HR, 2.38; 95% CI, 1.95-2.90) categories were larger compared with the full sample. A significant association was also observed in the overweight group, in contrast to the full sample. Kaplan-Meier curves (Figure 2D-F) revealed more spread among the groups than for all individuals. The upward trend of the HR seen in the full sample was preserved among never smokers and was also found for continuous maximum BMI (Table 2). These findings confirmed that smoking status was an important confounder when estimating the association between weight status and mortality.

Hazard ratios for baseline BMI in overweight (HR, 0.96; 95% CI, 0.89-1.04), obese I (HR, 1.15; 95% CI, 1.04-1.28), and obese II (HR, 1.65; 95% CI, 1.41-1.93) categories were lower than those for maximum BMI (eFigure 2 in the Supplement) in both the full sample and never smokers. Analyses using both maximum BMI and baseline BMI (eTable 6 in the Supplement) revealed that people who lost weight and fell into a different category after attaining their maximum BMI during the weight history period had the highest risk of mortality. For example, individuals who were once obese but were normal weight at baseline had an HR of 1.80 (95% CI, 1.23-2.64) compared with individuals who belonged to the normal-weight category all of the time.

**Figure 3.**
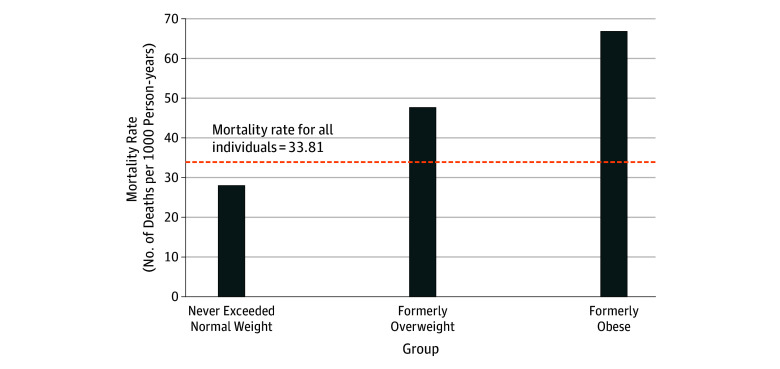
Mortality Rates for Individuals Who Were Normal Weight at Baseline, Stratified by Maximum Body Mass Index

In our study, individuals who never exceeded normal weight had a mortality rate of 27.93 per 1000 person-years (Figure 3). The mortality rates of normal-weight individuals who were formerly overweight or obese were 47.48 and 66.67 per 1000 person-years, respectively. Therefore, people who converted from overweight or obese to normal weight at baseline raised the mortality rate of the normal-weight category to 33.81 per 1000 person-years. This influx is the major reason why baseline BMI had lower HRs than maximum BMI, a typical representation of reverse causality.

### Cause-Specific Mortality

We performed secondary analyses using cause-specific mortality as our outcome (eTable 7 in the Supplement) on all individuals. Analyses for never smokers were not conducted because of a limited number of events. Cardiovascular disease mortality had larger risks with maximum BMI (HR, 1.22; 95% CI, 1.02-1.45 for overweight; HR, 1.66; 95% CI, 1.36-2.02 for obese I; and HR, 2.56; 95% CI, 2.00-3.28 for obese II) compared with other outcomes. However, there were significant associations between obesity and mortality due to other causes (HR, 1.21; 95% CI, 1.03-1.41 for obese I; HR, 1.93; 95% CI, 1.58-2.35 for obese II) and due to cancer (HR, 1.39; 95% CI, 1.06-1.84 for obese II).

## Discussion

In this study, we found a statistically significant monotonic association between maximum BMI over 24 years of weight history and all-cause mortality, with increasing risks observed across the obese I and obese II categories relative to normal weight. In contrast, estimates based on weight at a single point in time showed an apparent paradoxical association between overweight and mortality and attenuated associations with obese I and obese II, suggesting that reverse causality may have a prominent role in creating the obesity paradox. In analyses limiting the sample to never smokers, estimates strengthened further, with overweight emerging as a significant risk factor for mortality. Stratifying by sex, men had larger mortality risks than women.

Prior studies^1,2,3,4,5,22^ of mortality risks of obesity have typically used a single point in time to measure adiposity status. Our study findings indicate that failure to incorporate weight history may introduce substantial bias into assessment of risk. Specifically, in analyses stratified by weight history, mortality risks were found to be substantially higher in normal-weight individuals who had a history of overweight or obesity compared with those who maintained a normal-weight status over time. While maximum BMI restricts the definition of normal weight to those who maintained this status over time, in studies using single-point-in-time measures, the normal-weight category conflates the low-risk, stable-weight individuals with the high-risk reduced weight individuals, obscuring the substantial benefits associated with a normal-weight status.

Weight loss is consistently associated with elevated mortality risks in the observational literature.^23,24,25^ The elevated risks are likely explained by several factors, including illness-induced weight loss (reverse causality)^9,26^ and age-related changes in body composition, often referred to as sarcopenia, which lead to reduced skeletal muscle mass and bone mineral density.^27,28^ Although some weight loss is clearly voluntary, it is unlikely the major driver of the associations in the present study given compelling evidence that voluntary weight loss is associated with improved outcomes across a variety of end points,^29,30^ combined with the fact that voluntary weight loss is rare at the population level.^31^ A sensitivity analysis in our study revealed that individuals experiencing rapid weight loss had higher mortality risks than individuals experiencing slower weight loss, possibly indicative of the severity of an underlying disease. Regardless of the underlying mechanisms of weight loss in the present study, the fact that those who lost weight exhibited higher mortality risks in the present study reinforces the need to treat them separately from those who maintained normal weight across time, accomplished only by incorporating weight history.

Our analysis provides intriguing new evidence on the age pattern of the obesity-mortality association. Prior literature generally suggests that the mortality risks of obesity decline with age^6,32^; however, this result may simply reflect increasing reverse causal biases at older ages. Consistent with this hypothesis, we found a reversal in the age pattern of the BMI-mortality association (from a negative to a positive association) in models using maximum BMI vs baseline as the exposure variable. Potential explanations for the stronger association between obesity and mortality at older ages include the possibility of increased cumulative exposure to obesity with age, which has been demonstrated as an independent risk factor for all-cause mortality,^33^ and the onset of conditions with long latency periods, such as cancer.

Consistent with several prior studies,^6,34^ the results of the present study suggest that the association of obesity with all-cause and CVD-specific mortality may have declined over time. Prospective follow-up of the 2 cohorts included in our study spanned a period over which CVD mortality declined significantly in the United States.^35^ Prior evidence has attributed these declines to improvements in drug therapies, revascularization, acute care, risk factor control, and behavioral modification.^36,37,38^ Given that CVD is a major pathway through which obesity influences mortality, it is possible that improvements in CVD treatment and risk factor control have contributed to reductions in the risks associated with obesity.

### Strengths and Limitations

Our study has several important strengths. We incorporated data on the original and offspring cohorts of the FHS, which have long follow-up periods. The extensive follow-up on both cohorts made it possible to evaluate maximum BMI over 24 years of weight history, a substantially longer period than used in prior investigations of maximum BMI and mortality.^15^ Weight and height were directly measured at each participant’s examination visits, making it possible to obtain more robust estimates of maximum BMI than in prior studies that relied on recall data^14^ or self-reported longitudinal histories.^15^ Although several studies have investigated the validity of recall data and demonstrated close correspondence with measured BMI,^39,40^ self-reporting errors can bias estimates of the obesity-mortality association,^18,41^ and these biases may be exacerbated when BMI is categorized rather than treated continuously.^17^

Although the present study has strengths compared with the literature on maximum BMI and mortality, it has several limitations. First, there were 4- to 8-year intervals between adjacent examinations on FHS offspring cohort. As a result, we may have failed to capture true maximum BMI of some individuals if they reached their maximum BMI at a certain time point between 2 examinations. Second, our sample had few underweight participants, limiting our ability to investigate associations within that group. Third, most of the original and offspring cohorts were of white race/ethnicity, limiting the generalizability of the findings to other racial/ethnic groups. Fourth, collecting additional information on the intentionality of BMI change may help to elucidate the association further. Therefore, one potential future direction would be to take intentionality of weight loss into consideration.

## Conclusions

We found monotonically increasing risks of mortality across the obese I and obese II categories relative to normal weight using maximum BMI over 24 years of weight history. Incorporating weight history into studies of obesity and mortality could effectively reduce the consequences of reverse causation due to weight loss from illness. Therefore, eliciting weight history in clinical practice may be valuable for identifying patients at increased risk of mortality.

## Notes

Supplement. **eFigure 1.** Flowchart of Sample Exclusion **eFigure 2.** Hazard Ratios for Categories of Maximum BMI With 24 Years of Weight History and Baseline BMI Among Both All Individuals and Never Smokers **eTable 1.** Characteristics of Prior Conditions Among BMI Categories **eTable 2.** Analysis of Trends in the Mortality Risks Between FHS Original and Offspring Cohorts for Overweight and Obesity Categories of Maximum BMI **eTable 3.** Hazard Ratios for All-Cause Mortality in the FHS Original and Offspring Cohorts for Maximum BMI Categories With 24 Years of Weight History, Stratified by Smoking Status, Sex, and Cohort **eTable 4.** Hazard Ratios for All-Cause Mortality in the FHS Original and Offspring Cohorts for Maximum BMI Categories With 24 Years of Weight History and BMI Categories at Baseline Exam, Stratified by Age of 70 at Baseline **eTable 5.** Hazard Ratios for All-Cause Mortality in the FHS Original and Offspring Cohorts for Maximum BMI Categories With 24 Years of Weight History, Stratified by Smoking Status and Sex, Further Adjusting for BMI at Baseline Exam as Sensitivity Analyses **eTable 6.** Sample Size and Hazard Ratios for All-Cause Mortality for FHS Original and Offspring Cohorts Stratified by Maximum BMI and Baseline BMI Among All Individuals **eTable 7.** Hazard Ratios for Cause-Specific Mortality in the FHS Original and Offspring Cohorts for Maximum BMI Categories With 24 Years of Weight History
