# Erroneous Word in Methods Section

**DOI:** 10.1001/jamanetworkopen.2018.6657

**Published:** 2018-12-07

In the Original Investigation titled “Association of Obesity With Mortality Over 24 Years of Weight History: Findings From the Framingham Heart Study,”^1^ the word “age” was deleted from a sentence in the Sample Restriction subsection of the Methods because it changed the sentence’s meaning. The sentence now reads, “Participants were followed up to 42 years.” This article was corrected online.^1^
