# Error in Table 2

**DOI:** 10.1001/jamanetworkopen.2026.8540

**Published:** 2026-03-27

In the Original Investigation titled “Association of Obesity With Mortality Over 24 Years of Weight History: Findings From the Framingham Heart Study,”^1^ published November 16, 2018, in Table 2, the row label “Events/1000 person-years, No.” should have read “Person-years, No. (in thousands).” The article has been corrected.^1^
