# Supplementary material for: Association of Obesity With Mortality Over 24 Years of Weight History: Findings From the Framingham Heart Study
Source: JAMA Netw Open. 2018 Nov 16;1(7):e184587. doi: 10.1001/jamanetworkopen.2018.4587 (PMC6324399; doi:10.1001/jamanetworkopen.2018.4587)
Supplement: Supplement. — eFigure 1. Flowchart of Sample Exclusion eFigure 2. Hazard Ratios for Categories of Maximum BMI With 24 Years of Weight History and Baseline BMI Among Both All Individuals and Never Smokers eTable 1. Characteristics of Prior Conditions Among BMI Categories eTable 2. Analysis of Trends in the Mortality Risks Between FHS Original and Offspring Cohorts for Overweight and Obesity Categories of Maximum BMI eTable 3. Hazard Ratios for All-Cause Mortality in the FHS Original and Offspring Cohorts for Maximum BMI Categories With 24 Years of Weight History, Stratified by Smoking Status, Sex, and Cohort eTable 4. Hazard Ratios for All-Cause Mortality in the FHS Original and Offspring Cohorts for Maximum BMI Categories With 24 Years of Weight History and BMI Categories at Baseline Exam, Stratified by Age of 70 at Baseline eTable 5. Hazard Ratios for All-Cause Mortality in the FHS Original and Offspring Cohorts for Maximum BMI Categories With 24 Years of Weight History, Stratified by Smoking Status and Sex, Further Adjusting for BMI at Baseline Exam as Sensitivity Analyses eTable 6. Sample Size and Hazard Ratios for All-Cause Mortality for FHS Original and Offspring Cohorts Stratified by Maximum BMI and Baseline BMI Among All Individuals eTable 7. Hazard Ratios for Cause-Specific Mortality in the FHS Original and Offspring Cohorts for Maximum BMI Categories With 24 Years of Weight History [file jamanetwopen-e184587-s001.pdf]

Supplementary Online Content

Xu H, Cupples LA, Stokes A, Liu C-T. Association of obesity with mortality over 24 years of weight history: findings from the Framingham Heart Study. *JAMA Netw Open*. 2018;1(7):e184587. doi:10.1001/jamanetworkopen.2018.4587

eFigure 1. Flowchart of Sample Exclusion
eFigure 2. Hazard Ratios for Categories of Maximum BMI With 24 Years of Weight History and Baseline BMI Among Both All Individuals and Never Smokers
eTable 1. Characteristics of Prior Conditions Among BMI Categories
eTable 2. Analysis of Trends in the Mortality Risks Between FHS Original and Offspring Cohorts for Overweight and Obesity Categories of Maximum BMI
eTable 3. Hazard Ratios for All-Cause Mortality in the FHS Original and Offspring Cohorts for Maximum BMI Categories With 24 Years of Weight History, Stratified by Smoking Status, Sex, and Cohort
eTable 4. Hazard Ratios for All-Cause Mortality in the FHS Original and Offspring Cohorts for Maximum BMI Categories With 24 Years of Weight History and BMI Categories at Baseline Exam, Stratified by Age of 70 at Baseline
eTable 5. Hazard Ratios for All-Cause Mortality in the FHS Original and Offspring Cohorts for Maximum BMI Categories With 24 Years of Weight History, Stratified by Smoking Status and Sex, Further Adjusting for BMI at Baseline Exam as Sensitivity Analyses
eTable 6. Sample Size and Hazard Ratios for All-Cause Mortality for FHS Original and Offspring Cohorts Stratified by Maximum BMI and Baseline BMI Among All Individuals
eTable 7. Hazard Ratios for Cause-Specific Mortality in the FHS Original and Offspring Cohorts for Maximum BMI Categories With 24 Years of Weight History

This supplementary material has been provided by the authors to give readers additional information about their work.

© 2018 Xu H et al. *JAMA Network Open*.

eFigure 1. Flowchart of Sample Exclusion

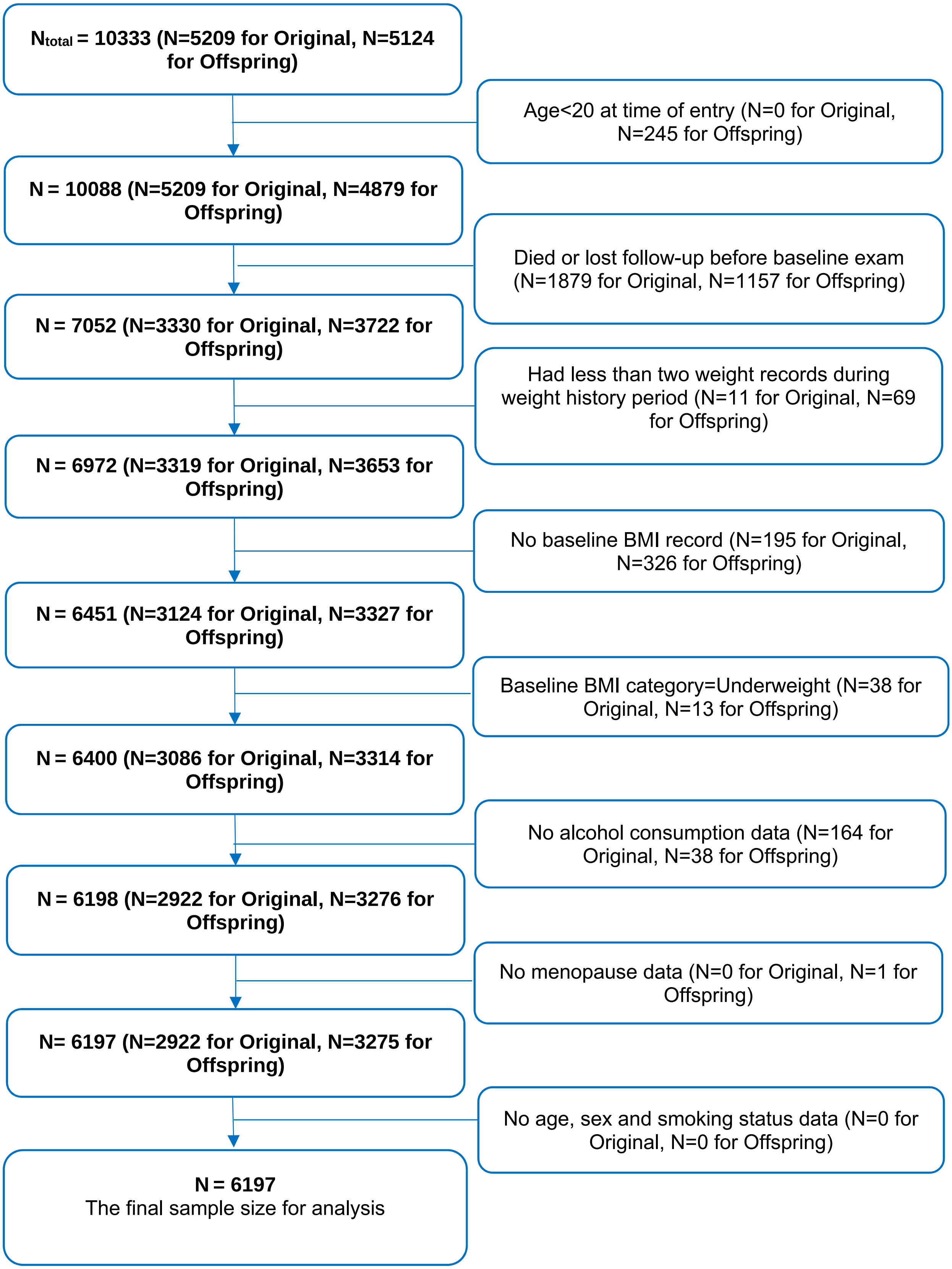

© 2018 Xu H et al. *JAMA Network Open*.

eFigure 2. Hazard Ratios for Categories of Maximum BMI With 24 Years of Weight History and Baseline BMI Among Both All Individuals and Never Smokers

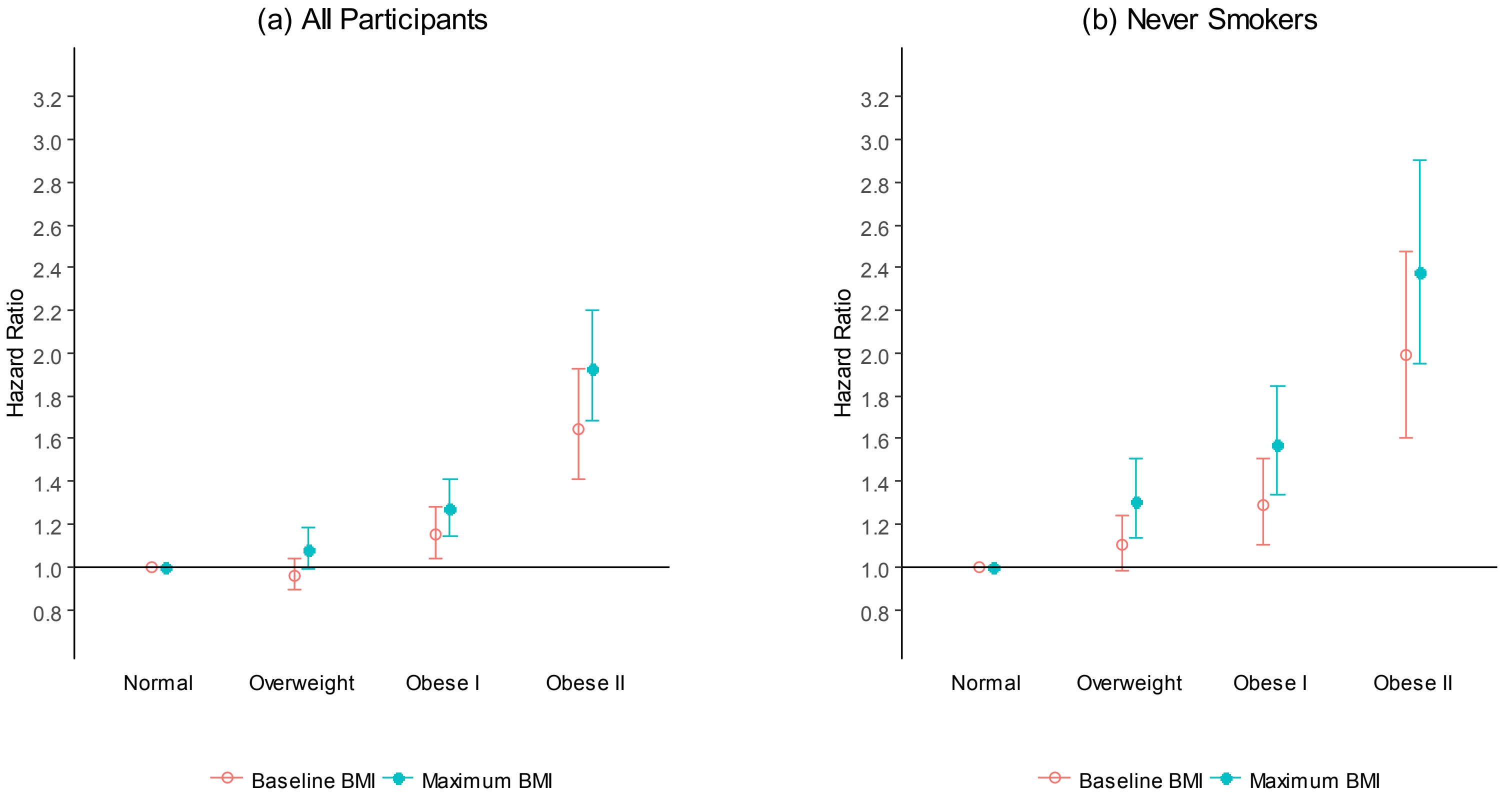

© 2018 Xu H et al. *JAMA Network Open.*

eTable 1. Characteristics of Prior Conditions Among BMI Categories

Maximum / Baseline BMI categories		All		Prior CVD				Prior cancer			
				No		Yes		No		Yes	
		N	%	N	%	N	%	N	%	N	%
**All**		6197	100	5765	93.0	432	7.0	5763	93.0	434	7.0
**Maximum BMI**	**Baseline BMI**										
Normal	Normal	1404	100.0	1339	95.4	65	4.6	1309	93.2	95	6.8
Overweight	Normal	642	100.0	579	90.2	63	9.8	576	89.7	66	10.3
Overweight	Overweight	2266	100.0	2130	94.0	136	6.0	2107	93.0	159	7.0
Obese	Normal	33	100.0	28	84.9	5	15.2	32	97.0	1	3.0
Obese	Overweight	423	100.0	371	87.7	52	12.3	388	91.7	35	8.3
Obese	Obese	1429	100.0	1318	92.2	111	7.8	1351	94.5	78	5.5

BMI categories: Normal (18.5-24.9 kg/m^2), Overweight (25.0-29.9 kg/m^2), Obese (≥30.0 kg/m^2)

© 2018 Xu H et al. *JAMA Network Open*.

eTable 2. Analysis of Trends in the Mortality Risks Between FHS Original and Offspring Cohorts for Overweight and Obesity Categories of Maximum BMI

**Strata**	**Overweight**	**Obese I**	**Obese II**
**All Individuals**			
Original	1 (Reference)	1 (Reference)	1 (Reference)
Offspring[a]	0.81 (0.66,0.99)	0.72 (0.57,0.91)	0.89 (0.67,1.18)
Male			
Original	1 (Reference)	1 (Reference)	1 (Reference)
Offspring	0.70 (0.50,0.97)	0.55 (0.39,0.79)	0.72 (0.45,1.15)
Female			
Original	1 (Reference)	1 (Reference)	1 (Reference)
Offspring	0.82 (0.62,1.09)	0.86 (0.62,1.18)	0.92 (0.64,1.32)
**Never Smokers**			
Original	1 (Reference)	1 (Reference)	1 (Reference)
Offspring	0.81 (0.59,1.11)	0.77 (0.54,1.09)	0.97 (0.64,1.46)
Male			
Original	1 (Reference)	1 (Reference)	1 (Reference)
Offspring	0.64 (0.31,1.12)	0.56 (0.30,1.04)	0.73 (0.34,1.57)
Female			
Original	1 (Reference)	1 (Reference)	1 (Reference)
Offspring	0.80 (0.53,1.21)	0.84 (0.53,1.33)	0.95 (0.57,1.58)

[a]: Multivariable model- including maximum BMI category, cohort, interaction term of maximum BMI category and cohort, together with other covariates (age at baseline, sex (if appropriate), smoking status (if appropriate), alcohol intake, menopause status (if appropriate), duration between exam of reaching maximum BMI and baseline exam) in the model

BMI categories: Normal (18.5-24.9 kg/m^2), Overweight (25.0-29.9 kg/m^2), Obese I(30.0-34.9 kg/m^2), Obese II(≥35 kg/m^2)

© 2018 Xu H et al. *JAMA Network Open*.

eTable 3. Hazard Ratios for All-Cause Mortality in the FHS Original and Offspring Cohorts for Maximum BMI Categories With 24 Years of Weight History, Stratified by Smoking Status, Sex, and Cohort

Strata	Original Cohort				Offspring Cohort			
	Normal	Overweight	Obese I	Obese II	Normal	Overweight	Obese I	Obese II
**All Individuals**								
Event (n)	532	1316	542	186	178	376	221	127
Multivariable HR (95% CI)[a]	1 (Reference)	1.14 (1.03,1.26)	1.37 (1.21,1.54)	1.91 (1.61,2.26)	1 (Reference)	0.91 (0.75,1.09)	0.98 (0.80,1.20)	1.80 (1.43,2.28)
Male								
Event (n)	151	671	280	56	63	252	138	60
Multivariable HR (95% CI)	1 (Reference)	1.25 (1.05,1.50)	1.58 (1.30,1.94)	2.43 (1.78,3.32)	1 (Reference)	0.89 (0.68,1.18)	0.91 (0.67,1.24)	2.00 (1.39,2.88)
Female								
Event (n)	381	645	262	130	115	124	83	67
Multivariable HR (95% CI)	1 (Reference)	1.10 (0.97,1.25)	1.28 (1.09,1.50)	1.78 (1.46,2.18)	1 (Reference)	0.90 (0.69,1.16)	1.08 (0.81,1.44)	1.64 (1.21,2.23)
**Never Smokers**								
Event (n)	179	548	249	96	79	209	106	68
Multivariable HR (95% CI)	1 (Reference)	1.39 (1.17,1.65)	1.69 (1.40,2.06)	2.30 (1.79,2.96)	1 (Reference)	1.12 (0.86,1.46)	1.33 (0.98,1.80)	2.45 (1.76,3.41)
Male								
Event (n)	33	217	88	21	24	142	65	32
Multivariable HR (95% CI)	1 (Reference)	2.05 (1.42,2.97)	2.59 (1.72,3.88)	3.83 (2.19,6.71)	1 (Reference)	1.35 (0.87,2.09)	1.55 (0.96,2.50)	3.34 (1.94,5.74)
Female								
Event (n)	146	331	161	75	55	67	41	36
Multivariable HR (95% CI)	1 (Reference)	1.24 (1.02,1.50)	1.50 (1.20,1.88)	2.01 (1.52,2.67)	1 (Reference)	0.98 (0.68,1.42)	1.25 (0.83,1.89)	1.97 (1.28,3.05)

[a]: Multivariable model- including maximum BMI category and other covariates (age at baseline, sex (if appropriate), smoking status (if appropriate), alcohol intake, menopause status (if appropriate), duration between exam of reaching maxbmi and baseline exam) in the model

BMI categories: Normal (18.5-24.9 kg/m^2), Overweight (25.0-29.9 kg/m^2), Obese I(30.0-34.9 kg/m^2), Obese II(≥35 kg/m^2)

Abbreviations: HR=Hazard Ratio, p-yrs=person-years, CI= Confidence Interval

© 2018 Xu H et al. *JAMA Network Open*.

eTable 4. Hazard Ratios for All-Cause Mortality in the FHS Original and Offspring Cohorts for Maximum BMI Categories With 24 Years of Weight History and BMI Categories at Baseline Exam, Stratified by Age of 70 at Baseline

Strata	Maximum BMI Category				Baseline BMI Category			
	Normal	Overweight	Obese I	Obese II	Normal	Overweight	Obese I	Obese II
**All Individuals**								
Age < 70 years								
Event (n)	519	1088	492	219	796	1004	372	146
Multivariable HR (95% CI)	1 (Reference)	1.00 (0.90,1.11)	1.20 (1.06,1.37)	1.92 (1.63,2.25)	1 (Reference)	0.96 (0.88,1.06)	1.23 (1.08,1.40)	1.95 (1.62,2.34)
Age ≥ 70 years								
Event (n)	191	604	271	94	414	527	174	45
Multivariable HR (95% CI)	1 (Reference)	1.32 (1.12,1.56)	1.48 (1.22,1.78)	2.06 (1.60,2.65)	1 (Reference)	1.10 (0.96,1.25)	1.19 (0.99,1.44)	1.51 (1.10,2.07)
**Never Smokers**								
Age < 70 years								
Event (n)	158	403	203	101	252	390	148	75
Multivariable HR (95% CI)	1 (Reference)	1.22 (1.01,1.47)	1.59 (1.29,1.96)	2.48 (1.93,3.20)	1 (Reference)	1.21 (1.03,1.42)	1.48 (1.20,1.82)	2.47 (1.89,3.23)
Age ≥ 70 years								
Event (n)	100	354	152	63	239	300	101	29
Multivariable HR (95% CI)	1 (Reference)	1.43 (1.14,1.80)	1.54 (1.19,1.99)	2.27 (1.65,3.12)	1 (Reference)	1.08 (0.91,1.29)	1.13 (0.89,1.44)	1.70 (1.14,2.53)

[a]: Multivariable model- including maximum BMI category (or Baseline BMI category) and other covariates (age at baseline, sex (if appropriate), smoking status (if appropriate), alcohol intake, menopause status (if appropriate), duration between exam of reaching maxbmi and baseline exam) in the model

BMI categories: Normal (18.5-24.9 kg/m^2), Overweight (25.0-29.9 kg/m^2), Obese I(30.0-34.9 kg/m^2), Obese II(≥35 kg/m^2)

Abbreviations: HR=Hazard Ratio, CI= Confidence Interval

© 2018 Xu H et al. *JAMA Network Open*.

eTable 5. Hazard Ratios for All-Cause Mortality in the FHS Original and Offspring Cohorts for Maximum BMI Categories With 24 Years of Weight History, Stratified by Smoking Status and Sex, Further Adjusting for BMI at Baseline Exam as Sensitivity Analyses

Strata	Maximum BMI Category			
	**Normal** (18.5-24.9 kg/m^2)	**Overweight** (25.0-29.9 kg/m^2)	**Obese I** (30.0-34.9 kg/m^2)	**Obese II** (≥35.0 kg/m^2)
**All Individuals**				
Event (n)<br>1,000 p-yrs	710<br>25.42	1692<br>49.63	763<br>21.88	313<br>8.28
Multivariable HR (95% CI)[a]	1 (Reference)	1.203 (1.08,1.34)	1.585 (1.345,1.867)	2.839 (2.195,3.671)
**Male**				
Event (n)<br>1,000 p-yrs	214<br>5.55	923<br>23.93	418<br>10.91	116<br>2.86
Multivariable HR (95% CI)	1 (Reference)	1.267 (1.061,1.514)	1.666 (1.282,2.165)	3.303 (2.186,4.989)
**Female**				
Event (n)<br>1,000 p-yrs	496<br>19.87	769<br>25.70	345<br>10.97	197<br>5.43
Multivariable HR (95% CI)	1 (Reference)	1.178 (1.025,1.353)	1.57 (1.265,1.948)	2.694 (1.931,3.758)
**Never Smokers**				
Event (n)<br>1,000 p-yrs	258<br>12.42	757<br>24.87	355<br>10.79	164<br>4.47
Multivariable HR (95% CI)	1 (Reference)	1.483 (1.252,1.756)	2.052 (1.598,2.634)	3.78 (2.575,5.549)
**Male**				
Event (n)<br>1,000 p-yrs	57<br>2.59	359<br>10.98	153<br>4.45	53<br>1.37
Multivariable HR (95% CI)	1 (Reference)	1.787 (1.302,2.454)	2.153 (1.382,3.354)	4.008 (2.063,7.785)
**Female**				
Event (n)<br>1,000 p-yrs	201<br>9.84	398<br>13.89	202<br>6.34	111<br>3.10
Multivariable HR (95% CI)	1 (Reference)	1.382 (1.125,1.697)	2.044 (1.498,2.789)	3.771 (2.343,6.068)

[a]: Multivariable model- including maximum BMI category and other covariates (age at baseline, cohort, sex (if appropriate), smoking status (if appropriate), alcohol intake, menopause status (if appropriate), duration between exam of reaching maximum BMI and baseline exam and BMI at baseline exam) in the model

Abbreviations: HR=Hazard Ratio, p-yrs=person-years, CI= Confidence Interval

© 2018 Xu H et al. *JAMA Network Open*.

eTable 6. Sample Size and Hazard Ratios for All-Cause Mortality for FHS Original and Offspring Cohorts Stratified by Maximum BMI and Baseline BMI Among All Individuals. (Obese I and Obese II were combined as Obese in this specific analysis due to small event size)

**Sample Size / Hazard Ratios (95% CI)**		**Maximum BMI**		
		Normal	Overweight	Obese
**Baseline BMI**	Normal	1404 / 1 (Reference)	642 / 1.22 (1.09, 1.38)	33 / 1.80 (1.23, 2.64)
	Overweight		2266 / 1.04 (0.94, 1.14)	423 / 1.33 (1.16, 1.53)
	Obese			1429 / 1.32 (1.18, 1.48)

BMI categories: Normal (18.5-24.9 kg/m^2), Overweight (25.0-29.9 kg/m^2), Obese (≥30.0 kg/m^2)

© 2018 Xu H et al. *JAMA Network Open*.

eTable 7. Hazard Ratios for Cause-Specific Mortality in the FHS Original and Offspring Cohorts for Maximum BMI Categories With 24 Years of Weight History

Strata	Normal	Overweight	Obese I	Obese II
**All-Cause mortality**				
Event (n) /1,000 p-yrs	710 / 25.42	1692 / 49.63	763 / 21.88	313 / 8.28
Multivariable HR (95% CI)[a]	1 (Reference)	1.08 (0.99,1.18)	1.27 (1.14,1.41)	1.93 (1.68,2.20)
**CVD**				
Event (n)	173	501	257	103
Multivariable HR (95% CI)	1 (Reference)	1.22 (1.02,1.45)	1.66 (1.36,2.02)	2.56 (2.00,3.28)
**Cancer**				
Event (n)	189	442	180	70
Multivariable HR (95% CI)	1 (Reference)	1.02 (0.85,1.21)	1.02 (0.82,1.25)	1.39 (1.06,1.84)
**Other Causes**				
Event (n)	348	749	326	140
Multivariable HR (95% CI)	1 (Reference)	1.05 (0.92,1.19)	1.21 (1.03,1.41)	1.93 (1.58,2.35)

[a]: Multivariable model- including maximum BMI category and other covariates (age at baseline, cohort, sex (if appropriate), smoking status, alcohol intake, menopause status (if appropriate), duration between exam of reaching maximum BMI and baseline exam) in the model

BMI categories: Normal (18.5-24.9 kg/m^2), Overweight (25.0-29.9 kg/m^2), Obese I(30.0-34.9 kg/m^2), Obese II(≥35 kg/m^2)

© 2018 Xu H et al. *JAMA Network Open.*
